# The Emerging Role of Altered d-Aspartate Metabolism in Schizophrenia: New Insights From Preclinical Models and Human Studies

**DOI:** 10.3389/fpsyt.2018.00559

**Published:** 2018-11-06

**Authors:** Francesco Errico, Tommaso Nuzzo, Massimo Carella, Alessandro Bertolino, Alessandro Usiello

**Affiliations:** ^1^Department of Agricultural Sciences, University of Naples “Federico II”, Portici, Italy; ^2^Translational Neuroscience Unit, IRCCS Casa Sollievo Della Sofferenza, San Giovanni Rotondo, Italy; ^3^Group of Psychiatric Neuroscience, Department of Basic Medical Science, Neuroscience and Sense Organs, Aldo Moro University, Bari, Italy; ^4^Laboratory of Behavioural Neuroscience, Ceinge Biotecnologie Avanzate, Naples, Italy; ^5^Department of Environmental, Biological and Pharmaceutical Sciences and Technologies, Università Degli Studi Della Campania “Luigi Vanvitelli”, Caserta, Italy

**Keywords:** d-aspartate, d-serine, schizophrenia, NMDA receptor, d-aspartate oxidase, mouse models

## Abstract

Besides d-serine, another d-amino acid with endogenous occurrence in the mammalian brain, d-aspartate, has been recently shown to influence NMDA receptor (NMDAR)-mediated transmission. d-aspartate is present in the brain at extracellular level in nanomolar concentrations, binds to the agonist site of NMDARs and activates this subclass of glutamate receptors. Along with its direct effect on NMDARs, d-aspartate can also evoke considerable l-glutamate release in specific brain areas through the presynaptic activation of NMDA, AMPA/kainate and mGlu5 receptors. d-aspartate is enriched in the embryonic brain of rodents and humans and its concentration strongly decreases after birth, due to the post-natal expression of the catabolising enzyme d-aspartate oxidase (DDO). Based on the hypothesis of NMDAR hypofunction in schizophrenia pathogenesis, recent preclinical and clinical studies suggested a relationship between perturbation of d-aspartate metabolism and this psychiatric disorder. Consistently, neurophysiological and behavioral characterization of *Ddo* knockout (*Ddo*^−/−^) and d-aspartate-treated mice highlighted that abnormally higher endogenous d-aspartate levels significantly increase NMDAR-mediated synaptic plasticity, neuronal spine density and memory. Remarkably, increased d-aspartate levels influence schizophrenia-like phenotypes in rodents, as indicated by improved fronto-hippocampal connectivity, attenuated prepulse inhibition deficits and reduced activation of neuronal circuitry induced by phencyclidine exposure. In healthy humans, a genetic polymorphism associated with reduced prefrontal *DDO* gene expression predicts changes in prefrontal phenotypes including greater gray matter volume and enhanced functional activity during working memory. Moreover, neurochemical detections in *post-mortem* brain of schizophrenia-affected patients have shown significantly reduced d-aspartate content in prefrontal regions, associated with increased *DDO* mRNA expression or DDO enzymatic activity. Overall, these findings suggest a possible involvement of dysregulated embryonic d-aspartate metabolism in schizophrenia pathophysiology and, in turn, highlight the potential use of free d-aspartate supplementation as a new add-on therapy for treating the cognitive symptoms of this mental illness.

## Introduction

Although l-amino acids are mostly used for protein synthesis and metabolic processes in eukaryotes, some free amino acids are present in a substantial amount in d configuration in mammalian tissues, including humans. In particular, free d-aspartate (d-Asp), and d-serine (d-Ser) are enriched in the brain where they emerge in an age- and region-dependent manner (1–5). Since its discovery at the beginning of the 90s (6), d-Ser has been extensively studied in the mammalian brain. Nowadays, it is well known that d-Ser is an endogenous *N*-Methyl d-Aspartate (NMDA) receptor (NMDAR) co-agonist that regulates the activation of glutamatergic excitatory synapses (7–10), thus influencing different NMDAR-dependent functions, such as brain development (11), synaptic transmission and plasticity (12–17), and behaviors (18–20). In this line, alteration of d-Ser metabolism has been linked to pathological conditions associated with NMDAR dysfunction, including schizophrenia (21–23). Unlike d-Ser, the neurobiological role of d-Asp has been most significantly studied only from the last decade, so it is still poorly defined. Nonetheless, we know today that d-Asp is able to directly modulate NMDAR-mediated transmission and functions in rodents, and dysregulation of its metabolism occurs in the brain of schizophrenia patients. In this review, we will summarize the evidence collected in both animal models and humans that have led to hypothesize a role for this atypical amino acid in schizophrenia pathophysiology.

## Presence of free d-aspartate and its metabolic regulation in the mammalian brain

The presence of free d-Asp in the mammalian brain was reported for the first time by Dunlop et al. in the mid-80s (1). This pioneering observation has been followed by many other studies in rodents and humans that overall showed a transient emergence of d-Asp in the brain, characterized by a peak of concentration during developmental stages and a drastic fall after birth (1–4, 24, 25). Among these studies, HPLC detections obtained by Hashimoto et al. revealed that the amount of d-Asp in the human prefrontal cortex (PFC) homogenates even exceeds that of the corresponding l-form at gestational week 14 (3). Consistent with this finding, in the rat brain d-Asp appears at embryonic day 12 (E12) selectively in non-proliferating neuroblasts but not in mitotic cells of the ventrocaudal forebrain, midbrain, and hindbrain (24). In the ventral side of the forebrain, d-Asp emerges in cell bodies of migrating neuroblasts and then shifts to axons once these cells have reached the outer layer of neural epithelium. Between E14 and E20, d-Asp concentration increases and spreads to the whole brain. In particular, in the cerebral cortex, d-Asp is present in both the cell bodies and projections of neuroblasts (24). In another work, Snyder et al. revealed that d-Asp is present in a substantial amount at birth in the forebrain regions, like the cortex and part of the midbrain (25). At post-natal day 2, d-Asp extends to the hindbrain and cerebellum and is enriched in the subventricular zone and the cortical plate of the cerebral cortex, the CA1-CA3 area and the dentate gyrus of the hippocampus, and the external granular layer of the cerebellum. In this last brain region, d-Asp staining is associated with granule cells that have not yet migrated to their definitive location (25). One week after birth, d-Asp decreases and almost disappears in one-month-old brains (26). Notably, in both prenatal and postnatal phases, d-Asp seems to be present exclusively in neurons but not in glial cells (25, 26).

**Figure 1:**
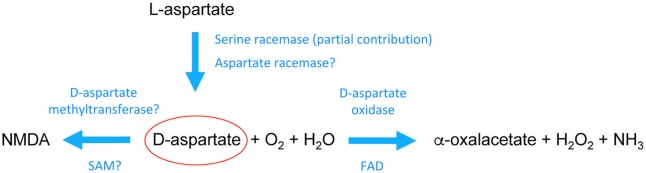
Schematic pathway of the biochemical transformations of d-aspartate in the mammalian brain. d-aspartate is likely generated through stereochemical inversion of l-aspartate. Recent studies in mice have shown that Serine racemase is partially involved in d-aspartate biosynthesis although it is still unclear whether its activity toward aspartate is region- and/or age-dependent. These studies also suggested that there should be a main racemase activity specifically involved in d-aspartate generation. On the other hand, it is acknowledged that d-aspartate is degraded by the enzyme d-aspartate oxidase through a process of deaminative oxidation (requiring flavin adenine dinucleotide, FAD, as a prosthetic group) that produces the ketoacid α-oxalacetate, hydrogen peroxide, and ammonia. In addition, d-aspartate can be converted into its N-methyl derivative, NMDA, possibly through a d-aspartate methyltransferase activity that uses s-adenosylmethionine (SAM) as a methyl donor. See the main text for further descriptions.

The peculiar spatiotemporal occurrence of d-Asp in the embryonic brain suggests that this atypical molecule is mainly produced through a *de novo* biosynthetic mechanism (Figure 1). Accordingly, a time-dependent accumulation of d-Asp was demonstrated in PC12 cells cultured in d-Asp-free medium (27, 28). Further evidence resulted from primary cultures of rat embryonic neurons in which [^14^C]-d-Asp was produced starting from [^14^C]-l-Asp precursor (25). A pyridoxal 5'-phosphate (PLP)-dependent glutamate-oxalacetate transaminase 1-like 1 (Got1l1) was identified as the enzyme that converts l-Asp to d-Asp in the adult mouse brain (29). However, subsequent findings have demonstrated that knockout of *Got1l1* gene does not produce any change in cerebral d-Asp levels of mutant mice (30). Interestingly, two studies have shown that serine racemase (SR), the only known enzyme responsible for d-Ser biosynthesis in the mammalian brain (31), could be involved also in d-Asp production, at least in some brain areas (32, 33). Indeed, *Sr* knockout mice display considerably decreased d-Asp levels in the forebrain regions (around 50–60 %) but not in the cerebellum (32, 33). Remarkably, overexpression of SR in rat PC12 cells resulted in intracellular d-Asp increase while genetic ablation of *Sr* in these cells did not produce changes in this d-amino acid levels (33), further supporting that besides SR an additional enzymatic activity for d-Asp biosynthesis is present in mammals.

While d-Asp biosynthetic pathway is still unclear, the enzyme responsible for d-Asp degradation, d-aspartate oxidase (DDO, or DAPOX EC 1.4.3.1), has been well known for a long time (34). DDO is a peroxisomal flavoenzyme (35, 36) that oxidizes d-Asp in presence of H_2_O and O_2_, producing α-oxaloacetate, H_2_O_2_, and ${\text{NH}}_{4}^{+}$ (Figure 1) (37–39). DDO is inactive toward d-Ser (40) that is degraded by its homologous flavoenzyme, d-amino acid oxidase (DAAO, EC 1.4.3.3) (41–43). A specific tripeptide signal at the C-terminus of DDO targets this enzyme to peroxisomes (40, 44), where the toxic H_2_O_2_ produced by d-Asp oxidation can be safely removed by peroxisome-resident catalases (45).

Consistent with a primary role for DDO activity in controlling its substrate concentration, it has been reported that spatial and temporal expression of this enzyme is reciprocal to d-Asp (26, 35, 46). During mouse lifespan, the postnatal decrease of cerebral d-Asp is mirrored by a time-dependent increase of *Ddo* gene expression, as *Ddo* mRNA levels are very low from E14 to 1 week of age and then strongly rise during the following weeks until adult phase (46). Interestingly, the postnatal increase of *Ddo* transcript levels is temporally associated with concomitant dramatic demethylation of the putative promoter region of *Ddo* gene. In agreement with an influence of DNA methylation on *Ddo* mRNA expression, it has been shown that treatment with the DNA demethylating agent, azacitidine, triggers the expression of *Ddo* transcript in embryonic cortical neurons, which do not express *Ddo* gene in physiological conditions (46). Notably, the temporal pattern of *Ddo* mRNA expression reported in the mouse brain matches closely with the substantial increase in DDO enzymatic activity found in the rat brain during the first weeks of life (35). Like d-Asp, also DDO is prominently localized in neurons, and only marginally expressed in glial population (36). Overall, reciprocal spatiotemporal localization of DDO and d-Asp suggests that during adulthood this enzyme is necessary to remove its endogenous substrate from brain regions where d-Asp is no more required and, indeed, could be functionally detrimental and neurotoxic for neuronal activity and survival, as found in the brain of elderly constitutive knockout mice for *Ddo* gene (*Ddo*^−/−^) (46–48). The substantial and persistent accumulation of d-Asp in the brain of *Ddo*^−/−^ mice further suggests that DDO is the only enzyme that catalyzes the endogenous degradation of d-Asp, throughout the entire animal lifespan (47, 49, 50).

## Pharmacological features of d-aspartate

Pioneering neuropharmacological studies performed in the second half of the 80s, aimed at finding molecules with agonistic or antagonistic activity for l-glutamate (l-Glu) receptors, highlighted that d-Asp is able to bind to the l-Glu site of NMDARs (51–55). In particular, one of these works revealed that the efficiency of d-Asp in displacing the NMDAR antagonist, d-AP5, in rat brain membranes is the same as NMDA and around 10 times lower than l-Glu (54). Many years later, a series of electrophysiological observations demonstrated that d-Asp not only binds to NMDARs but also activates these receptors. Indeed, local application of d-Asp on adult mouse brain slices triggers inward currents in both hippocampal CA1 pyramidal neurons and striatal GABAergic medium spiny neurons, which are strongly reduced by the competitive and non-competitive NMDAR antagonists, d-AP5 and MK-801, respectively (56, 57). d-Asp activates NMDARs through the binding to each of the GluN2 subunits (58), and induces the transient increase of intracellular Ca^2+^ (47). Notably, a small but significant percentage of the currents triggered by d-Asp application still endure even after application of high doses of MK-801 (47, 56, 58). The persistence of d-Asp-mediated currents under conditions of NMDAR blockade suggests that this d-amino acid may be able to affect, at least in part, additional receptor complexes. Accordingly, d-Asp can also stimulate metabotropic mGlu5 receptors in mouse and rat brain (59–61).

The endogenous occurrence of d-Asp in the brain and its main ability to activate NMDARs are consistent with an involvement of this d-amino acid in the *in vivo* modulation of glutamatergic synaptic functioning. In agreement with this idea, early studies have suggested that d-Asp can transit through the extracellular space by transporter mechanisms enabling its release and reuptake. Accordingly, experiments using different *ex vivo* preparations (tissue slices, cells or synaptosomes) demonstrated that d-Asp is released through exocytotic processes mediated by vesicular Ca^2+^ (25, 62–65). d-Asp is contained in membrane-bound organelles, identified as secretory granules (in PC12 cells) (64) or synaptic vesicles (in synaptosome preparation) (66), in which this d-amino acid is actively stored through still unknown transporter systems. In line with *in vitro* data, Usiello et al. confirmed the existence of Ca^2+^-dependent d-Asp release in the cortex of living mice. Indeed, in microdialysis studies performed in the PFC of freely moving animals they showed that d-Asp is present in the extracellular space at nanomolar concentration (46, 61) and when dialysates were collected in Ca^2+^-free artificial cerebrospinal fluid, extracellular concentration of d-Asp dropped below the limit of HPLC detection. Extracellular d-Asp has been also recently demonstrated in the brain of domestic chicks, where it decreases in an age-dependent manner and is transiently induced following high K^+^ stimuli (67). The recent evidence that d-Asp occurs also in the extracellular space confirms the idea that this d-amino acid has the potential to bind to NMDARs also *in vivo* and, in turn, to affect the glutamatergic neurotransmission dependent on this receptor subclass. In addition to Ca^2+^-dependent efflux system, further studies reported that d-Asp may also be released through spontaneous processes (25, 68, 69) or mechanisms involving l-Glu exchange (70, 71).

Different studies suggest that intracellular uptake of d-Asp may depend by l-Glu/l-Asp transporter system, which moves excitatory amino acids against their concentration gradient using the Na^+^/K^+^ electrochemical gradient. Interestingly, while on one side this carrier system recognizes only the l-enantiomer of Glu, on the other it is able to take up both Asp enantiomers with approximately the same efficiency (72). In line with an involvement of L-Glu/L-Asp transporters in the reuptake of d-Asp, autoradiographic and immunostaining experiments have shown that d-Asp preloading on rodent hippocampal and cerebellar slices causes a strong labeling in glutamatergic axons and in surrounding glial processes (73–75). No direct evidence is so far available about the existence of selective d-Asp reuptake mechanisms in the *in vivo* brain. Nonetheless, the drastic and fast reduction of cortical extracellular d-Asp after experimental removal of Ca^2+^ from the artificial cerebrospinal fluid reported in microdialysis studies (46, 61), seems to suggest the presence of an active and efficient mechanism of d-Asp clearance in the mammalian brain.

## Preclinical models with a non-physiological increase of d-aspartate content in the brain

The generation of *Ddo* knockout mice (*Ddo*^−/−^) in two independent laboratories (49, 50) has been a turning point for the comprehension of the *in vivo* role of d-Asp and its catabolizing enzyme. Both *Ddo*^−/−^ mouse lines display higher d-Asp levels in the brain and peripheral organs, but comparable l-Asp (49, 50) and l-Glu content (50), compared to their respective controls. In more detail, HPLC detection in homogenates deriving from the hippocampus, striatum, cortex, cerebellum and olfactory bulbs showed ~10- to 20-fold increase in d-Asp levels in *Ddo*^−/−^ mice, compared to wild-type animals (46, 47, 56, 57). Along with d-Asp, also the levels of the N-methyl derivative of d-Asp, NMDA, were increased in *Ddo*^−/−^ brain homogenates (47, 49). In this regard, biochemical findings indicated that cerebral NMDA could be generated through the transfer of a methyl group from S-adenosyl methionine to d-aspartate catalyzed by a methyltransferase activity (Figure 1) (76). In line with the rise of total d-Asp levels in brain homogenates, recent evidence on PFC dialysates has indicated that *Ddo*^−/−^ mice display significantly increased d-Asp concentration also in the extracellular fraction, compared to *Ddo*^+/+^ littermates (a rise of about five times, ~100 nM in *Ddo*^−/−^ vs. ~20 nM in *Ddo*^+/+^) (46).

Besides *Ddo* gene targeting, another experimental approach has been widely used in the last years to raise the cerebral levels of d-Asp. This approach consists of the oral administration of d-Asp to animals for periods of one-two months until the age of two-three months. In relation to the brain region analyzed, such a treatment produces an approximately 2- to 5-fold increase in d-Asp content in homogenate samples, compared to the same areas of untreated mice (56–58, 61). Such rise is substantially lesser than that produced by *Ddo* gene targeting since the DDO enzyme expressed in the brain of adult d-Asp-treated mice is able to limit the further increase of this d-amino acid levels. In line with the effect of *Ddo* gene ablation, also the exogenous administration of d-Asp is able to induce a significant extracellular increase of this molecule in the PFC of freely moving mice, in both chronic and acute conditions (61). In particular, chronic oral administration of d-Asp for 1 month produces an increase in the same order of magnitude as *Ddo* knockout (~80 nM in treated mice vs. ~20 nM in untreated mice). On the other hand, acute d-Asp injection (at the dose of 500 mg/kg) is able to trigger a transient and rapid elevation of d-Asp levels already 20 min post-administration (from ~20 to ~500 nM). Consistent with the observations of Usiello et al. (61), a very recent study showed that intragastric infusion of d-Asp to rats reaches the hippocampus in 15 min via blood circulation (77). Taken together, these findings have a potential translational relevance as they highlight the ability of d-Asp to cross the blood-brain barrier, as described so far only for the d-stereoisomers of Ser and proline (78, 79). Strikingly, both chronic oral administration and acute injection of d-Asp are able to evoke a significant prefrontal efflux of l-Glu (48, 61) through the presynaptic activation of NMDA, mGlu5, and AMPA/kainate receptors triggered by this d-amino acid (61). Overall, these data indicate that d-Asp could influence glutamatergic neurotransmission not only through its direct binding to the l-Glu site of postsynaptic NMDARs but also through its ability to evoke the presynaptic release of endogenous l-Glu in selective brain regions.

## d-aspartate affects functional and structural neuronal properties dependent on NMDA receptors activation

The results described so far suggest that d-Asp stimulates also *in vivo* the glutamatergic transmission in adult rodents. In this regard, electrophysiological evidence obtained in the last 10 years has revealed that higher d-Asp content enhances NMDAR-dependent early-phase and late-phase long-term potentiation (E-LTP and L-LTP, respectively) in the CA1 area of the hippocampus of both adult *Ddo*^−/−^ and chronically d-Asp-treated mice (47, 56, 58, 80). Interestingly, the induction of E-LTP protocol, which causes a decaying LTP in *Ddo*^+/+^ and untreated mice, is sufficient to maintain an enduring L-LTP (lasting for more than 160 min following tetanic stimulation) in *Ddo*^−/−^ and d-Asp-treated mice (80). In both models, d-Asp-dependent L-LTP is insensitive to rapamycin administration but is suppressed by cytochalasin D (80), a potent inhibitor of actin polymerization. Moreover, chronic treatment with d-Asp increases the frequency of NMDAR-mediated miniature excitatory post-synaptic currents in pyramidal neurons of the medial PFC layer II/III (80). Remarkably, in line with the enhanced glutamatergic transmission, mice chronically treated with d-Asp display stronger metabolic activity in fronto-hippocampal areas, measured by basal cerebral blood volume-weighted functional magnetic resonance imaging (fMRI) (80). In this regard, increased synchronization between the hippocampus and cortical regions has been also recently produced through d-Asp gavage in awake rats subjected to blood oxygen level dependent (BOLD) fMRI (77). Changes in synaptic functioning are commonly associated with structural synaptic variations in dendritic morphology (81, 82). This relationship is observed also in the brain of *Ddo*^−/−^ and d-Asp-treated mice, in which facilitated induction of late-phase synaptic plasticity is mirrored by increased dendritic length and spine density, and greater dendritic arborisation in pyramidal neurons of the PFC and hippocampus (80). These *in vivo* results are inferred also from very recent *in vitro* findings showing that the exogenous application of d-Asp to rat hippocampal slices produces a rapid genesis of middle size spines of CA1 neurons via an actin-sensitive mechanism, as early as 2 h after treatment (77). Consistent with structural and functional enhancement in cortico-hippocampal synaptic plasticity, *Ddo*^−/−^ and d-Asp treated mice display significant improvements in the cognitive domain of spatial memory when tested in the hidden-platform version of the Morris water maze and contextual fear conditioning (47, 57), two behavioral tasks involving the hippocampal activation of NMDARs (83, 84). Similar behavioral observations were reported also in d-Asp-treated rats (85).

However, if on the one hand NMDARs promote synaptic strength and connectivity, on the other they can produce neuronal death if their stimulation is abnormally intense or temporarily too long (86). In line with detrimental NMDAR-related effects, persistent increase of d-Asp levels results in precocious decay of basal glutamatergic transmission, synaptic plasticity and hippocampal reference memory in 13/14-month-old *Ddo*^−/−^ (47), mirrored by loss of excitatory glutamatergic synapses and reduction of synaptic GluN1 and GluN2B subunits (87). In addition, a recent study also revealed that the lack of DDO leads to severe neuroinflammation processes and cell death in an age-dependent manner (46). In line with the results obtained in elderly *Ddo*^−/−^ animals, also aged C57BL/6J mice treated with d-Asp (at the dose of 20 mM) for 12 months display similar deficits in hippocampal NMDAR-mediated synaptic plasticity despite such prolonged administration results in only two-fold d-Asp increase in this brain region (58). However, it should be noted that the interruption of d-Asp treatment for 3 weeks is sufficient to fully rescue the NMDAR-dependent LTP deficits and that such a long administration schedule does not produce locomotor and anxiety-like alterations in aged animals that, on the contrary, display a significant cognitive improvement in cue-dependent fear conditioning paradigm (58). Overall, these data point out that the “Yin and Yang” behavior of NMDARs (86) can be recapitulated in different stages of life by deficient DDO activity in the mouse brain.

## d-aspartate affects schizophrenia-related features and activation of neuronal circuits in the rodent brain

Accumulating evidence supports the hypothesis that a developmental hypofunction of NMDARs is a causative factor in the etiology and pathophysiology of schizophrenia (88–91). In the wake of the glutamatergic model of schizophrenia, several clinical, pharmacological, imaging, and genetic findings suggest today that dysfunctional metabolism of the NMDAR co-agonist, d-Ser, may produce NMDAR-mediated alterations leading to the manifestation of this mental illness (21–23, 92).

In the last years, a number of preclinical observations have shown that also d-Asp may contribute to influence some NMDAR-dependent phenotypes related to schizophrenia. For instance, behavioral studies performed in adult *Ddo*^−/−^ and d-Asp-treated mice have revealed that chronic d-Asp elevation significantly reduces the prepulse inhibition (PPI) deficit induced by psychotomimetic drugs like amphetamine and MK-801 without affecting basal properties of sensorimotor filtering (57). In addition, *Ddo*^−/−^ mice display a substantial reduction of motor hyperactivity, ataxia and PPI disruption triggered by acute administration of phencyclidine (PCP) (93, 94), the drug that better than any other models schizophrenia symptoms in both humans and rodents (22, 95–97). In line with behavioral observations, increased levels of d-Asp in *Ddo*^−/−^ mice are also able to counteract the dysfunctional cortico-limbo-thalamic activation induced by PCP, as measured by fMRI (94). Moreover, consistent with increased dendritic length and spine density, resting-state fMRI has shown greater cortico-hippocampal connectivity in the brain of *Ddo*^−/−^ mice (94). Increased functional connectivity between the hippocampus and cortex has been recently found also in the rat brain following intragastric administration of d-Asp (77). Since several clinical and preclinical studies suggest the occurrence of cortico-hippocampal dysconnectivity in schizophrenia (98, 99), the *in vivo* imaging data obtained in animal models with higher d-Asp levels highlight a potential translational relevance for d-Asp in treating this mental illness. Consistently, increased levels of d-Asp are also able to prevent corticostriatal long-term depression (57), a synaptic feature reported also in mice chronically treated with the typical antipsychotic haloperidol (100).

## Reduced d-aspartate concentration in the human prefrontal cortex of schizophrenia *post-mortem* brain

Based on the results obtained in preclinical research, recent studies have focused the attention on humans in order to assess d-Asp metabolism and its impact on phenotypes relevant to schizophrenia. So far, two different studies have measured d-Asp levels in two different cohorts of *post-mortem* brain samples deriving from patients with schizophrenia and corresponding non-psychiatric controls (101, 102). The first study, performed on a small number of samples (7–10 subjects/diagnosis), revealed reduced d-Asp levels (about 40%) in the *post-mortem* PFC of schizophrenia-affected patients (101), linked to significantly increased *DDO* mRNA levels in the same brain area (94). However, neither methylation changes in the putative *DDO* promoter nor gross aberrations in this gene, including insertion, deletion, frameshift, or nonsense mutations were found in the same samples (94). Also the second study, performed on a larger number of samples (20 subjects/diagnosis/brain region), reported a significant d-Asp reduction (about 30%) selectively in the dorsolateral PFC (DLPFC) but not in the hippocampus of patients, compared to the respective brain regions of non-psychiatric subjects (102). Interestingly, the biochemical analysis pointed out that reduced content of d-Asp in the DLPFC of schizophrenia-affected subjects is associated with an aberrant increase in DDO enzymatic activity (102). However, differently from the first cohort of samples (101), *DDO* gene expression, as well as DNA methylation status, was comparable between diagnoses (102, 103). Moreover, western blotting analysis of SR, regarded as an enzyme involved in d-Asp biosynthesis (32, 33), revealed no changes between schizophrenia-affected patients and healthy individuals (102).

Furthermore, to assess the effect of putative alteration of d-Asp metabolism in the human brain, another study reported the association among *DDO* gene variations, prefrontal *DDO* mRNA expression and structural/functional prefrontal phenotypes relevant to schizophrenia (80). Within this study, an *in silico* analysis performed on 268 *post-mortem* brains of non-psychiatric subjects (deriving from BrainCloud bank, http://braincloud.jhmi.edu) revealed that the C allele of the single nucleotide polymorphism (SNP), rs3757351, mapping in an intronic region of *DDO* gene, is associated with reduced prefrontal expression of *DDO* transcript, compared to the T allele and, thus, may hypothetically predict increased endogenous d-Asp levels in the PFC. This result led authors to perform *in vivo* imaging analyses whose results disclosed that healthy individuals bearing the C allele also display increased prefrontal gray matter volume and greater prefrontal activity, compared to the subjects with the T allele, when they were subjected to 1- and 2-Back working memory tasks.

Besides the neurochemical and functional studies reported above in *post-mortem* schizophrenia brain, a recent preclinical research by Usiello et al. revealed that the modulation of d-Asp metabolism could be instrumental for the mechanism of action by which a common second-generation antipsychotic, olanzapine, influences glutamatergic cortical neurotransmission (61). In particular, they found that olanzapine, differently from other typical and atypical antipsychotics, inhibits the enzymatic activity of DDO *in vitro*. Moreover, in agreement to *in vitro* results, chronic administration of this antipsychotic is able to increase the extracellular levels of d-Asp and l-Glu in the PFC of freely moving *Ddo*^+/+^ mice, but not in *Ddo*^−/−^ animals (61).

## Conclusions and future perspectives

The findings described in the present review highlight that increased levels of the endogenous NMDAR agonist, d-Asp, impact on a series of functional, and structural phenotypes relevant to schizophrenia. Despite the knowledge on the neurobiological role of d-Asp and its potential involvement in schizophrenia is significantly enhanced in the last years, many issues remain still unsolved or deserve further clarification. For instance, it seems clear today that d-Asp is synthesized in the brain where it is subjected to release and reuptake mechanisms that enable its neuromodulatory activity, but it is still unclear what are the specific enzymes and transport systems responsible for such processes. Is there a selective enzymatic activity responsible for d-Asp production in the mammalian brain? Moreover, the influence of SR upon the endogenous d-aspartate level still waits to be clarified in the human brain. If SR is actually involved in d-Asp biosynthesis in the human brain, to what extent it contributes to the generation of this d-amino acid and in which spatiotemporal window? Are there relevant genetic *SR* variants associated to dysregulation of cerebral d-Asp levels? These questions may help to elucidate the effective significance of altered d-Asp metabolism in schizophrenia since *SR* is in the list of the largest genome-wide association study as a susceptibility gene for schizophrenia (92). The assessment of a d-Asp involvement in schizophrenia will necessarily undergo the verification of altered d-Asp amount in further schizophrenia brains. Indeed, the results of a dysregulated d-Asp metabolism in *post-mortem* brain samples so far achieved are encouraging but refer only to a limited number of samples, analyzed by the same research team (101, 102). Therefore, this issue deserves to be further assessed also by other laboratories and in a larger number of *post-mortem* brain tissues as well as in other biological materials such as the peripheral blood and cerebrospinal fluid of patients (FE, AB and AU personal communication).

Another important aim that should be pursued in future studies concerns the evaluation of the potential therapeutic value of d-Asp in schizophrenia treatment. In support of its translational application, d-Asp is nowadays approved for the use in humans, and commercialized as a dietary supplement. Accordingly, recent studies have shown that d-Asp treatment has no toxicological consequences or influences on hormonal activity of the hypothalamic-pituitary-gonadal axis and the mass of skeletal muscle (104–108). Besides d-Asp administration, future therapeutic strategies may be disclosed by the identification of compounds with inhibitory activity against DDO and, thus, able to indirectly enhance the availability of cerebral d-Asp. Novel DDO inhibitors have been so far functionally assayed *in vitro* by Homma et al. (109) and await further *in vivo* characterization to test their clinical validity.

Finally, future research needs to provide clear evidence of the potential role of embryonic d-Asp metabolism in the pathophysiological mechanisms leading to schizophrenia. In this regard, it is very important to underline that the etiology of this psychiatric disorder is likely to involve genetic and environmental risk factors emerging during the neurodevelopmental stages (110–113), in coincidence with the peak of d-Asp, and affecting several processes controlled by NMDARs, such as neurogenesis, survival, migration and formation of brain circuits (114–116). Therefore, we speculate that dysfunctional d-Asp metabolism occurring during neurodevelopment may affect early critical processes dependent on NMDARs and, in turn, contribute to schizophrenia vulnerability. The recent generation of a genetic mouse model with prenatal reduction of d-Asp levels (FE and AU personal communication) may aid to disclose the importance of the transient occurrence of d-Asp in developmental brain processes and, in turn, the potential involvement of dysregulated d-Asp metabolism in a neurodevelopmental psychiatric disorder like schizophrenia.

## Author contributions

FE and AU conceived and wrote the manuscript. TN, MC and AB critically reviewed the manuscript.

### Conflict of interest statement

The authors declare that the research was conducted in the absence of any commercial or financial relationships that could be construed as a potential conflict of interest.
